# Bisphosphonates Targeting Ion Channels and Musculoskeletal Effects

**DOI:** 10.3389/fphar.2022.837534

**Published:** 2022-03-15

**Authors:** Rosa Scala, Fatima Maqoud, Marina Antonacci, Jacopo Raffaele Dibenedetto, Maria Grazia Perrone, Antonio Scilimati, Karen Castillo, Ramón Latorre, Diana Conte, Saïd Bendahhou, Domenico Tricarico

**Affiliations:** ^1^ Section of Pharmacology, Department of Pharmacy-Pharmaceutical Sciences, University of Bari, Bari, Italy; ^2^ Medicinal Chemistry Section, Department of Pharmacy-Pharmaceutical Sciences, University of Bari, Bari, Italy; ^3^ Centro Interdisciplinario de Neurociencia de Valparaíso, Facultad de Ciencias, Universidad de Valparaíso, Valparaíso, Chile; ^4^ Centro de Investigación de Estudios Avanzados, Universidad Católica del Maule, Talca, Chile; ^5^ UMR7370 CNRS, Laboratoire de Physiomédecine Moléculaire (LP2M), Labex ICST, Nice, France

**Keywords:** ATP sensitive potassium channel, TRPV1 channel, skeletal muscle, bone, bisphosphonate

## Abstract

Bisphosphonates (BPs) are the most used bone-specific anti-resorptive agents, often chosen as first-line therapy in several bone diseases characterized by an imbalance between osteoblast-mediated bone production and osteoclast-mediated bone resorption. BPs target the farnesyl pyrophosphate synthase (FPPS) in osteoclasts, reducing bone resorption. Lately, there has been an increasing interest in BPs direct pro-survival/pro-mineralizing properties in osteoblasts and their pain-relieving effects. Even so, molecular targets involved in these effects appear now largely elusive. Ion channels are emerging players in bone homeostasis. Nevertheless, the effects of BPs on these proteins have been poorly described. Here we reviewed the actions of BPs on ion channels in musculoskeletal cells. In particular, the TRPV1 channel is essential for osteoblastogenesis. Since it is involved in bone pain sensation, TRPV1 is a possible alternative target of BPs. Ion channels are emerging targets and anti-target for bisphosphonates. Zoledronic acid can be the first selective musculoskeletal and vascular KATP channel blocker targeting with high affinity the inward rectifier channels Kir6.1-SUR2B and Kir6.2-SUR2A. The action of this drug against the overactive mutants of *KCNJ9-ABCC9* genes observed in the Cantu’ Syndrome (CS) may improve the appropriate prescription in those CS patients affected by musculoskeletal disorders such as bone fracture and bone frailty.

## Introduction

Bisphosphonates (BPs) are the most used bone-specific anti-resorptive agents. With the Food and drug administration (FDA) approval of alendronate more than 3 decades ago, BPs were introduced in clinical practice (Kennel and Drake, 2009). BPs have been used as primary therapy for skeletal disorders caused by imbalanced bone homeostasis, where osteoblast and osteoclast activities are not perfectly coupled, leading to excessive bone resorption. Data obtained from several well-designed clinical trials clearly show that BPs are highly effective in reducing loss of bone mass, deterioration of bone microarchitecture, and increased fracture risk associated with aging, thereby increasing patients’ quality of life (Drake et al., 2008). Initially, BPs were not meant to be therapeutical drugs but were synthesized as anticorrosive and complexing agents. They were first produced in 1865 in Germany (Menschutkin, 1865) and used to produce fertilizers, textiles, and oil. Moreover, because of their capability to mask calcium ions preventing the precipitation of calcium carbonate, they were used as water softeners (Graham and Russell, 2011). Pharmacological properties of BPs were discovered later during an investigation about the mechanism of calcification and the role of pyrophosphate (Fleish and Neuman, 1961).

Polyphosphates can act as natural regulators of the calcification process because they inhibit the deposition of calcium salts. In particular, the presence of pyrophosphate (PPi) was postulated in body fluids, like plasma and urine. PPi was able to act as a calcification inhibitor by binding the crystals of hydroxyapatite (HA) and directly inhibiting the production of HA in the extracellular fluid (Fleisch and Bisaz, 1962). Moreover, because of their capability to mask calcium ions preventing the precipitation of calcium carbonate, they were used as water softeners (Graham and Russell, 2011). Pharmacological properties of BPs were discovered later, during an investigation about the mechanism of calcification and the role of pyrophosphate (Fleish and Neuman, 1961).

Therefore, it was hypothesized that the bone mineralization process could be regulated by altering the level of PPi (Russell 1965; Russell et al., 1971). Research on the therapeutic use of pyrophosphate revealed its efficacy only when administrated via direct injection. Oral administration leads to rapid hydrolysis of the compound in the gastrointestinal tract. The problem of PPi hydrolysis was solved by modifying the PPi structure until the final production of the BPs. Apart from regulating calcification even after oral administration, BPs can impede HA crystals dissolution (Fleisch et al., 1970; Russell et al., 1999). These advantages allowed the first use of BPs for human treatment with bone preservation purposes (Kuźnik et al., 2020).

### Structure-Activity Relationship of Bisphosphonates

**FIGURE 1:**
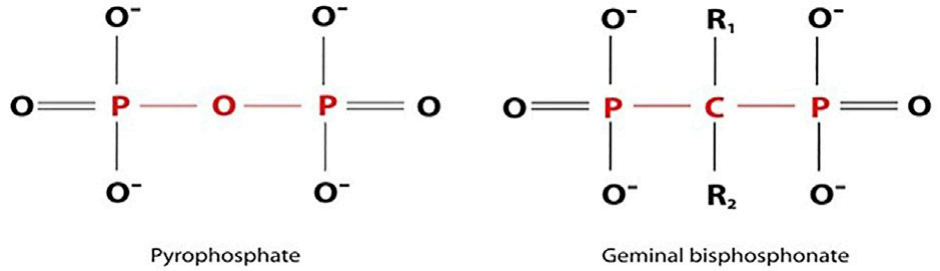
A structural analogy of bisphosphonates and pyrophosphates groups.

BPs are synthetic analogues of PPi, showing two phosphate groups binding to a central carbon atom. This typical P-C-P moiety makes the BPs resistant to breakdown by enzymatic hydrolysis, differently from the hydrolytically nonstable P-O-P bond in the structure of PPi (Figure 1).

BPs are generally divided into three groups according to their different structural characteristics and anti-resorptive potencies (Matsumoto et al., 2016). First Generation: BPs of this class, including etidronate and clodronate, are simple non-nitrogen-containing compounds. All the drugs show the typical P-C-P moiety but differ for the R1 and R2 groups (Figure 1). Second Generation: drugs show P-C-P moiety, where R1 is an–OH group and R2 contains alkylamine moieties. Alendronate and pamidronate belong to this group Third Generation: These are the most recently synthesized drugs, including risedronate and zoledronic acid, which have a P-C-P skeleton and R1 is–OH group, and R2 has nitrogen-containing heterocyclic moieties, responsible for the increased anti-resorptive activity.

**FIGURE 2:**
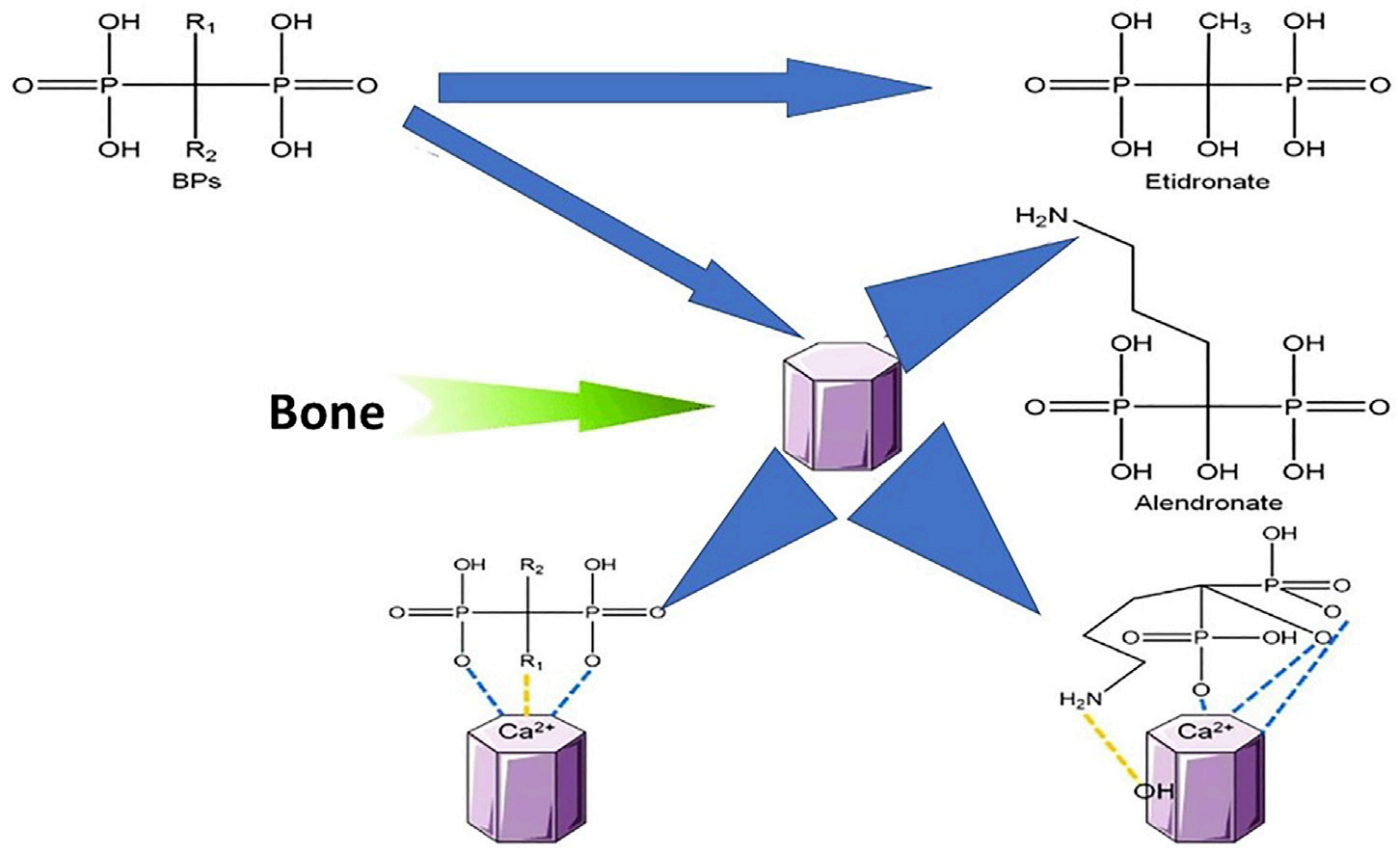
Structure and binding mechanisms of nitrogen non-containing (etidronate) and containing (alendronate) bisphosphonates (BPs) with bone hydroxyapatite matrix.

The P-C-P moiety responsible for the strong affinity to HA, with the two phosphate groups in close vicinity chelating the calcium ions on the surface of HA crystals and acting as a bidentate chelator (Iafisco et al., 2008). Moreover, the side chains R1 and R2 attached to the central carbon atom contribute to the biological properties of the drugs (Widler et al., 2002; Graham and Russell, 2011). For example, when R1 is an–OH group, BPs’ capability of binding to the mineral surface and preventing the growth and dissolution of HA crystals is increased thanks to the formation of a tridentate bond (Figure 2) (Shinoda et al., 1983; Russell et al., 2008). R2 determines the anti-resorptive potency of BPs and contributes to the HA affinity. Drugs with nitrogen in the R2 group show a higher affinity for HA (Lawson et al., 2010; Graham and Russell, 2011) and increased potency of 10–10.000 times relative to the non-nitrogen BPs (Dunford et al., 2001; Ding et al., 2018). A positive charge in the R2 facilitates the interaction with HA, consequently increasing bone affinity for the negatively charged phosphonate groups, thanks to electrostatic interactions (Russell et al., 2008). Moreover, amino groups of BPs may be involved in forming additional hydrogen bonds with the HA surface, again increasing the affinity for the bone tissue (Bosco et al., 2015) (Figure 2).

As expected by the high affinity toward HA, BPs are primarily taken and adsorbed into bone to exert their pharmacological effects. The human and animal data show that approximately one to two-thirds of the administered BPs doses becomes incorporated into the skeleton, and the remainder is excreted in the urine predominantly within the first few hours after administration (Russell et al., 2008) with a body clearance of 0.5L/h (ZOMETA^®^). The urinary excretion of small amounts of BPs can be measured over many weeks, months, or years after stopping treatment, presumably due to their ongoing release from the skeleton (Cremers et al., 2002; Russell et al., 2008). BPs distribution in bone is not homogenous but occurs predominantly in bone regions with high turnover. Macro-anatomically, bone can be divided into two different types: compact and trabecular bone. Compact, or cortical, bone is present in the middle portion, called diaphysis, of long bones and on the surface of the flat bones. Trabecular, or cancellous, bone is instead present in the long bones’ epiphyseal and metaphyseal regions (joints). Remodelling of the bone occurs differently in the two bone types, being much more significant in trabecular bone than compact ones. Therefore, the uptake of BPs in femur and tibia joints is much higher than in the central shaft (Carnevale et al., 2000), with a difference from two-to threefold (Leyvraz et al., 1992). These findings strongly support the notion that only the exposed HA in the resorption sites is available and accessible to the circulating BPs, thereby explaining the non-uniform distribution of BPs in bone. It should be noted that blood supply to trabecular bone is higher than to compact bone. In addition, the ratio between trabecular surface and volume is around 4:1 in trabecular bones compared with compact bones. These additional elements may contribute to the higher drug concentration in bone joints. Furthermore, bone uptake may also be affected by age and gender. Bone remodelling is an age-dependent process. Experiments performed on rats revealed a higher uptake of alendronate in young rats (2 months) compared with old ones (12 and 20 months), whereas no differences were observed between 12- and 20-month-old rats (Lin et al., 1992). In addition, BPs concentration was lower in young female rats compared to young male rats, but no differences were found among the two groups in adult age. BPs binding with HA in bone is saturable, supporting the idea that the binding occurs in specific and accessible sites (Lin, 1996).

Even though the bone is the main distribution site, BPs can also go to soft noncalcified tissues, like the liver, lung, kidney, and spleen (Russell et al., 2008). The noncalcified tissue/plasma concentration ratio is very low, between 0.05 and 0.7. However, in the kidney, which is involved in drug excretion, it may reach 6. BPs concentration in noncalcified tissue rapidly decreases with time, declining from 63% after 5 min from the administration of the drug to 5% after 1 h. On the contrary, drug concentration in bone increases continuously, reaching its peak after 1 h from the dose. This rapid redistribution of the BPs toward bones suggests that soft tissues contact the drug for a very short time. This interaction may explain, the direct effects of BPs on tumour cells or some of the non-bone-related side effects, such as muscle tissues (Clézardin, 2011; Clézardin et al., 2011; Maqoud et al., 2021).

It is not a surprise that the most frequently observed Adverse Drug Reactions (A.D.R.) of these drugs reported per System Organ Class (S.O.C.) were musculoskeletal and connective tissues disorders, general disorders, administration site conditions, and gastrointestinal disorders in the EudraVigilance database. In the case of zoledronic acid among musculoskeletal and connective tissue disorders, osteonecrosis of the jaw was the most frequently reported A.D.R., representing 47.9% of the total A.D.R. and affecting 4,792 females 3,184 males of all ages.. Arthralgia and myalgia were also frequently reported representing 16 and 10% of the A.D.R., respectively, with several unresolved cases. Muscular weakness was 2.7% of the A.D.R. per S.O.C., and some cases of atrophy and rhabdomyolysis with blood creatine phosphokinase increased were reported in the EudraVigilance database up to May 2021 (Maqoud et al., 2021). Severe suppression of bone turnover has been reported in association with alendronate therapy. In 2005, 9 patients taking alendronate sustained atypical fractures, associated with delayed healing (Odvina et al., 2005). Histomorphometry analysis of the cancellous bone in these patients revealed marked bone formation suppression, with reduced or absent osteoblastic surface and reduced extracellular matrix production. Instead, the osteoclastic surface was found low or low-normal with a consequent reduction in the eroded surface. The osteoclastic surface was instead found low or low-normal with a consequent reduction in the eroded surface. These findings raise the concern that long-term BPs therapy may lead to severe suppression of bone turnover, leading to impaired ability to repair skeletal microfractures and skeletal fragility. It should be noted that cases like the one reported here are difficult to associate with patient care because of the extremely high variance in terms of doses of BPs. Other simultaneous medications possibly affect bone or other undiagnosed skeletal abnormalities not related to the BPs therapy (Kennel and Drake, 2009).

Around 15 years ago, several cases of unusual fractures in the subtrochanteric region and along the femoral diaphysis in BPs-treated patients were reported (Odvina et al., 2005). These reports were followed by many other studies describing these same fractures, now called atypical femur fractures. Several cases of BPs-associated atypical femur fractures have been described over the years, although they remain an uncommon phenomenon. These fractures occur primarily in the proximal or mid femoral diaphysis, either spontaneously or resulting from low-energy trauma. In addition, they can be transverse or oblique (≤30°) with breaking of the cortex and diffuse cortical thickening of the proximal femoral shaft defined as a “simple with thick cortices” pattern. Patients with this pattern often manifest cortical thickening in the contralateral femur (Bolhofner, 2008). Generally, these fractures occur more frequently in patients receiving prolonged BPs therapy and are often associated with prodromal symptoms like thigh pain, vague discomfort, or subjective weakness (Kwek et al., 2008).

The association between atypical femur fractures and BPs has been questioned. Even if some studies showed minimal risk, it is now recognized the existence of a clear association, especially in the case of prolonged drug use (Black et al., 2020).

In a recent study analysing a large, prospective cohort of around 200 000 BP-treated women older than 50, 277 atypical femur fractures were found (1.74 fractures per 10.000 patients-years) (Black et al., 2020). In particular, there were 50 atypical femur fractures among women who were on a BP for 3 to less than 5 years (n = 29.287), a 9-fold higher risk than those who took BPs for less than 3 months (hazard ratio 8.86, 95% CI 2.79–28.20). Moreover, there were 95 atypical femur fractures among women taking a BP for 8 or more years (n = 16.893), around 43-fold increased risk (HR 43.51, 95% CI 13.70–138.15). The incidence of fracture was higher in the presence of risk factors, like Asian ancestry, shorter height, higher weight, and glucocorticoid use for 1 year or more. On the contrary, it rapidly decreases after drug discontinuation.

### Pharmacodynamics of Bisphosphonates: Osteoclast-Osteoblast Functional Coupling and Bone Homeostasis

BPs are drugs commonly used to treat of all the skeletal pathological conditions characterized by an imbalance between osteoclast-mediated bone resorption and osteoblast-mediated bone formation, including metastatic bone disease. Usually, osteoclast and osteoblast activities are highly coupled to preserve bone homeostasis. Bone is a very dynamic tissue, in a constant state of remodeling, attempting to fix all the micro-injuries that generally occur in life. During this remodeling process, some areas of bone are resorbed by osteoclasts and replaced by new bony tissue produced by osteoblasts (Grabowski, 2015). Even if highly coupled, osteoclast and osteoblast activities occur at a different rate. Orchestrating this process requires several factors, including local factors, such as growth factors, cytokines, and chemokines, and systemic factors, like the estrogens. The three main cells in this process are osteoclasts, osteoblasts, and osteocytes, whose related activities strictly depend on the previously cited regulating factors.

Osteoclasts are multinucleated giant cells that arise from the fusion of osteoclast precursor cells belonging to the hematopoietic stem cells located in the bone marrow like monocyte/macrophage lineage cells. Osteoclast precursor cells can be differentiated into osteoclasts in the presence of two typical factors: the receptor for macrophage/monocyte colony-stimulating factor (M-CSF) and the receptor activator of nuclear factor-κB (NF-κB) ligand (RANKL), both produced by osteoblasts (Grabowski, 2015). M-CSF induces on the pre-osteoclast membrane the receptor RANK for RANKL, allowing osteoclastogenesis and activating intracellular pathways essential for osteoclasts’ full maturation. In addition to these two pro-maturation factors, osteoblasts can also produce osteoprotegerin (OPG) receptor, which acts as a negative regulator of osteoclast maturation. OPG is a soluble decoy receptor for RANKL, it prevents its interaction with the RANK receptor. Resorption is associated with the secretion of acid and proteolytic enzymes, like the tartrate-resistant acid phosphatase (TRACP), finally dissolving both the organic collagen and the inorganic HA of the bone. Osteoclast-mediated bone resorption is a pH-depending process and requires high proton concentration and low pH.

The bone formation process is instead mediated by osteoblasts, responsible for maintaining bone density and volume. Osteoblasts, and the osteoblast-derived bone-lining cells and osteocytes, derive from a multipotent precursor of mesenchymal origin, named mesenchymal stem cells (MSC), which also give origin to several other cells such as chondrocytes, adipocytes, myocytes, and fibroblasts (Oreffo et al., 2005). Differentiation of MSCs into osteochondral progenitor is orchestrated by several Runx2 (Runt-related transcription factor 2) and Osx (Osterix zinc finger containing transcription factor) that are known to play a key role in osteoblast differentiation.

At the cellular level, BPs can inhibit bone resorption by selective binding the bone mineral surface. Here, BPs can be internalized into osteoclasts, interfere with their biochemical processes, and induce apoptosis. At the molecular level, the biochemical mechanisms of action differ among the BPs of the first generation and the nitrogen-containing BPs.

Early non-nitrogen-containing bisphosphonates, such as etidronate, clodronate, and tiludronate, are closely similar to PPi, so they can mimic its structure and function. First, there is osteoclast-mediated uptake of the drugs from the bone mineral surface; then, thanks to class II aminoacyl-transfer RNA synthases, they can be incorporated into molecules of newly formed adenosine triphosphate (ATP) with the formation of AppCH2p. This metabolite analog of ATP has P-C-P moiety in place of the β,γ pyrophosphate (P-O-P) moiety of ATP. In this way, it leads to non-hydrolysable (AppCp) nucleotides (Xu et al., 2013). Consequently, they accumulate in the cells generating cytotoxic effects on osteoclasts and causing a deficiency of functional ATPs, altering several ATP-dependent cellular processes, inhibiting the mitochondrial ADP/ATP translocase, and finally leading to osteoclast apoptosis.

**FIGURE 3:**
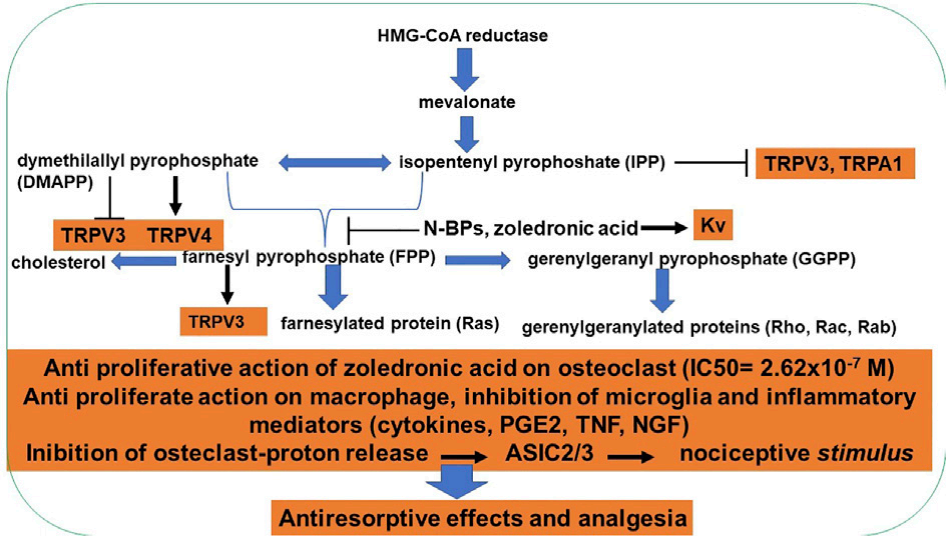
Schematic diagram of the mechanism of action of second and third BPs on osteoclasts. Nitrogen-containing BPs can inhibit FPP synthase, preventing the synthesis of FPP and GGPP required for the prenylation of proteins that are crucial for osteoclast function and survival. Inhibition of FPP synthase also causes the accumulation of IPP, which is incorporated into the cytotoxic metabolite. The action of intermediate metabolites on TRP channels. Consequences of inhibition of farnesyl diphosphate synthase by N-BPs on the nociceptive pathways.

Differently, the second and third generation drugs, such as alendronate, risedronate, ibandronate, pamidronate, and zoledronic acid, are nitrogen-containing BPs showing a different mechanism of action. Indeed, they can bind and inhibit the farnesyl pyrophosphate synthase (FPPS), the key enzyme of the mevalonate pathway, and weakly the geranyl pyrophosphate synthase (Soki et al., 2013). The first enzyme is essential to produce two five-carbon building blocks called isopentenyl pyrophosphate (IPP) and dimethylallyl pyrophosphate (DMAPP), which are used to make isoprenoids, such as cholesterol, heme, steroid hormones, vitamin K, and coenzyme Q10. In osteoclasts, two intermediates of the pathway, FPP (farnesyl pyrophosphate) and GGPP (geranylgeranyl pyrophosphate) have an essential role. These are required for the post-translational prenylation of small G-proteins, such as Ras, Rab, Rho, and Rac, which are prenylated at a cysteine residue. They regulate several osteoclast processes essential for their maturation and survival, like stress fiber assembly and membrane ruffling (Boanini et al., 2012). So, if GTPases are not produced, the formation of the ruffled border is inhibited, as well as the activity of lysosomal enzymes and the transcytosis of degraded bone matrix. The inhibition of FPPS leads to a loss of resorption capacity of osteoclasts and the inhibition of osteoclastogenesis. The intermediate metabolites IPP, DMAPP, and FPP modulate TRP channels with nociceptive effects (Tzschentke, 2019) (Figure 3).

BPs may exert anti-nociceptive, anti-allodynic, and anti-hyperalgesia properties against a large variety of noxious stimuli, like chemical, tactile or thermal, but are also plenty effective in spontaneous pain. Benefits have been reported in animal models of acute, inflammatory, and neuropathic pain, osteoarthritis, and osteoporosis. In addition, several case reports, open-label trials, or other exploratory studies report the analgesic effects of BPs in various diseases (Tzschentke, 2019).

Even so, the molecular targets involved in this secondary pharmacological property of BPs are not well understood. Therefore, despite the high potential of BPs in pain control, this lack of a well-defined mechanism of action limits the appreciation of BPs in the pain field (Tzschentke, 2019). Several putative analgesic mechanisms have been proposed for explaining the analgesic effects of BPs (Bang et al., 2012; Yao et al., 2016; Dohke et al., 2017; Tzschentke, 2019).

Analgesia may be a direct consequence of the “canonical” mechanism of action of BPs, namely the inhibition of FPPS in osteoclasts. In situations of high bone turnover, the increased activity of osteoclasts leads to the release of protons and acidification of the extracellular microenvironment. Protons are commonly known to activate acid-sensing ion channels (ASICs) expressed in the primary afferent sensory neurons, thereby inducing a nociceptive stimulus. Therefore, it is likely that BPs inhibition of osteoclasts activity may reduce the extracellular acidification avoiding the activation of primary nociceptive afferents located in bone (Dohke et al., 2017).

Furthermore, FPPS inhibition via BPs leads to the accumulation of the normal reagents it acts on, which are isopentenyl diphosphate (IPP) and dimethylallyl diphosphate (DMAPP) and prevents the formation of farnesyl diphosphate (FPP). DMAPP can act as a TRPV3 ion channel antagonist, promoting anti-nociceptive effects (Bang et al., 2012). In contrast, FPP is an agonist of the same channel acting as a pro-nociceptive molecule (Bang et al., 2012). Therefore, the inhibition of FPPS causes increased levels of anti-nociceptive molecules and decreased levels of pro-nociceptive molecules, providing a further explanation for the BPs analgesic properties. Even so, the causal relationship between FPPS inhibition and analgesia remains to be unequivocally demonstrated (Tzschentke, 2019).

Apart from the “canonical” and primary target of BPs, spinal microglial activation has also been proposed as a pathway involved in the analgesic effects of BPs. Alendronate can inhibit CD45, reducing the spinal microglia activation and p38 phosphorylation in the chronic constriction injury neuropathy model, reducing allodynia and hyperalgesia, and preventing the release of pro-inflammatory cytokines (Yao et al., 2016). Other markers of central sensitization have been proposed. Ibandronate was shown to reduce prodynorphin levels in a mouse cancer model normalizing its levels in the spinal cord and inducing analgesia (Halvorson et al., 2008). How the e. v. administration of BPs “*in vivo*” may allow the drug to penetrate the blood-brain barrier, reach the central nervous system and interact with the microglia remains to be explained.

BPs may act on the purinergic system, acting as antagonists of the P2X2/3 receptors. BPs may act on the purinergic system, acting as antagonists of the P2X2/3 receptors. P2X2/3 receptors are expressed in nociceptive sensory neurons and bone tissue, so involvement in bone pain related to osteoporosis can be hypothesized. Thus, the ubiquitous expression of these receptors in the peripheral tissues, including, but not limited to, skin, viscera, and the musculoskeletal system gives reason to consider the purinergic system as a potential analgesic target for BPs independently of their actions on osteoclasts and FPPS (Burnstock, 2013). Vesicular nucleotide transport (VNUT) has been also implicated in the analgesic properties of BPs. Their inhibition prevents the loading of synaptic vesicles with ATP, disrupting the synaptic release of ATP, finally interrupting the purinergic nociceptive transmission, and leading to analgesic and anti-inflammatory effects (Shima et al., 2016).

Despite osteoclast-mediated bone resorption having been considered the major pathway disrupted by BPs drugs, the involvement of only osteoclasts cannot explain all the pharmacological effects of BPs on bone mass. Alternative pharmacological targets should be involved. Bone formation coupled to bone resorption should also be decreased due to BPs therapy, resulting in an overall reduction in the rate of bone remodeling. Even so, BPs capability to reduce the incidence of bone fracture does not support this theory. Explaining the shift in the balance toward bone production it has been proposed that BPs may also directly affect osteoblasts activity. Therefore, in the last years, several studies have tried to explain this dichotomy by demonstrating that part of the beneficial effects of BPs on the skeleton is the prevention of osteoblast and osteocyte apoptosis, prolonging their lifespan and inducing mineralization. *In vivo* administration of BPs promotes early osteoblastogenesis in mice (Giuliani et al., 1998) and increases the expression of osteoblast differentiation markers. Humans treated with zoledronic acid show increased mineral apposition rate, consistently with the increased pro-mineralizing activity of the osteoblasts (Recker et al., 2008; Gnant et al., 2019). These data suggest that BPs positively affect bone formation, even in the face of reduced overall bone remodeling. These positive effects on osteoblast survival are exerted via different mechanisms apart from inhibiting the mevalonate pathway and producing toxic metabolites inside the osteoclasts. However, the exact molecular pathways involved in the effects on osteoblasts are unknown; and different hypotheses have been proposed.

Early studies showed that treating murine osteoblastic cells with BPs inhibits the differentiation of bone marrow or spleen osteoclasts precursors into mature osteoclasts (Nishikawa et al., 1996). Consistent with this early finding, BPs have been shown to decrease the expression of the receptor activator of NF-kB ligand (RANKL) and increase the expression of the RANKL decoy receptor osteoprotegerin (OPG) in human osteoblastic cells (Viereck et al., 2002). So, this theory suggests that the osteoblasts mediated anti-resorptive effects of BPs can be caused by the interference with the pro-differentiation and survival of osteoclasts induced by RANKL signaling (Boyce and Xing, 2008). Some studies have suggested that BPs such as alendronate and risedronate can prevent osteoblast and osteocyte apoptosis, either *in vitro* or *in vivo*, thus increasing the number of osteoblasts in the site of bone formation and their function (Plotkin et al., 1999; Follet et al., 2007; Bellido and Plotkin, 2011).

The first evidence of an ion channel involvement in BPs actions in osteoblast came from the study of Plotkin et al., 2002 who showed that BPs were able to modify connexin (Cx)43 permeability, thereby causing ERK activation. Cx43 is one of the major members of the connexin family of proteins expressed in osteoblasts and osteocytes. Connexin hexamers form hemichannels, which are plasma membrane channels. Hemichannels of adjacent cells can form gap junction channels that allow intercellular communication among osteocytes and osteoblasts. Clusters of channels can open transiently, permitting the exchange of small water-soluble molecules (<1 kDa). Also, they are localized in unopposed cell membranes causing the exchange of cytoplasmatic contents with the extracellular fluid. Opening of Cx43 hemichannels leads to the activation of kinases such as Src and ERKs (extracellular signaling-regulated kinases) that initiate sequential phosphorylation of the cytoplasmic target of the ERKs, p90RSK kinase, and final target substrates, BAD and the CCAAT/enhancer-binding protein C/EBPβ. Phosphorylation of BAD renders it inactive, whereas phosphorylation of C/EBPβ leads to the binding of pro-caspases, thus inhibiting apoptosis. The presence of Cx43 on the osteoblast membrane was found as a necessary condition for the induction of cell survival by BPs since alendronate was not able to prevent osteoblasts apoptosis in mice lacking Cx43 (Plotkin et al., 2002). No other connexins were instead required for the pro-survival effects of BPs (Bellido and Plotkin, 2011). It should be noticed that whether BPS, which show a small molecular size, enter osteoblastic cells after inducing hemichannels opening remains unknown. Moreover, BPs can bind cells even lacking Cx43 suggesting that the drugs bind to another moiety that subsequently can interact with Cx43. The identity of this protein is still unknown.

It is interesting to note that although these studies show that BPs can increase proliferation and stimulate differentiation on osteoblasts, others reveal that BPs decrease proliferation, also inhibiting osteoblast differentiation and mineralization (Kellinsalmi et al., 2005). This apparent contradiction is explained considering that pro-survival and pro-differentiation effects are exerted at very low concentrations, in a range from 10^−9^ M to 10^−6^ M (Tenenbaum et al., 1992). However, the anti-proliferative effects can be observed at higher concentrations, over 10^−5^ M (Kellinsalmi et al., 2005). The anti-apoptotic effects are exerted at BPs concentrations almost 3 orders of magnitude lower pro-apoptotic ones. In our hands, we found that BPs and novel structural related analogs were capable to inhibit cell proliferation of cancerous cells, murine osteoclast cells, and osteoclast precursors cells at 50–100 × 10^−6^ M concentrations, while inducing cell proliferation and mineralization of pre-osteoblastic like cell line MC3T3-E1 and murine osteoblast bone marrow-derived cells in the nanomolar concentration range (30–50 × 10^−9^M) after 10–15 days of incubation of the cells with the mineralization medium (Table 1) (Savino et al., 2018; Scala et al., 2019) (Table 1).

**TABLE 1:** Percentage changes of cell proliferation and mineralization with Bis-phosphonates in non-malignant and malignant cells.

Cells	Zoledronic acid concentrations 10 × 10^−9^M-500 × 10^−6^M	Alendronate concentrations 10 × 10^−9^M-500 × 10^−6^M	Risendronate concentrations 10 × 10^−9^M-500 × 10^−6^M
Murine osteoblast-like cells MC3T3-E115 after 72 h of incubation in DMEM + or 10–15 days of incubation in mineralization medium	30–50 × 10^−9^M = +90–140% changes of cell mineralization (*p* < 0.05)	30–50 × 10^−9^M = +50–55% changes of cell mineralization (*p* < 0.05)	30–50 × 10^−9^M = +55–60% changes of cell mineralization (*p* < 0.05)
	IC_50_ = 2 × 10^−5^M proliferation	IC_50_ = 10 × 10^−6^M proliferation	IC_50_ = 1.98 × 10^−5^M proliferation
Bone marrow cells from WT/WT mice after 72 h of incubation in DMEM + or 10–15 days of incubation in mineralization medium	10–50 × 10^−9^M = +18 ± 6% (n.s) proliferation	±20% changes of cell proliferation (n.s.)	±20% changes of cell proliferation (n.s.)
	30–50 × 10^−9^M = +30–38% mineralization (*p* < 0.05)	30–50 × 10^−9^M = +9 ± 4% changes of cell mineralization (n.s.)	30–50 × 10^−9^M = +15 ± 9% changes of cell mineralization (n.s.)
	100 × 10^−9^M:+26 ± 8% (*p* < 0.05) proliferation	—	—
	10 × 10^−6^M = -9 ± 4% (n.s) proliferation	—	—
	100–500 × 10^−6^M: 28 ± 5% (*p* < 0.05) proliferation	—	—
Human osteosarcoma cells MG63 after 72 h of incubation in DMEM+	10–50 × 10^−6^M: 22 ± 10% (n.s.) proliferation	10–50 × 10^−6^M: 17 ± 11% (n.s.) proliferation	10–50 × 10^−6^M: 11 ± 9% (n.s.) proliferation
	10^−4^M = -65 ± 10% (*p* < 0.05) proliferation	10^−4^M = -51 ± 6% (*p* < 0.05) proliferation	10^−4^M = -19 ± 7% (n.s.) proliferation
Murine pre-osteoclasts like cells RAW264.7 after 72 h of incubation in DMEM+	IC_50_ = 2.6 × 10^−7^M proliferation	IC_50_ = 5.9 × 10^−8^M proliferation	IC_50_ = 5.3 × 10^−7^M proliferation
	10^−4^M = -77 ± 6% (*p* < 0.05) proliferation	10^−4^M = −60 ± 6% (*p* < 0.05) proliferation	10^−4^M = −69 ± 9% (*p* < 0.05) proliferation
Murine pre-osteoclasts like cells J774A.1 after 72 h of incubation in DMEM+	10–50 × 10^−6^M: 18 ± 8% (n.s.) proliferation	10–50 × 10^−6^M: 14 ± 6% (n.s.) proliferation	10–50 × 10^−6^M: 5 ± 1% (n.s.) proliferation
	10^−4^M = -78 ± 4% (*p* < 0.05) proliferation	10^−4^M = -81 ± 7% (*p* < 0.05) proliferation	10^−4^M = -85 ± 4% (*p* < 0.05) proliferation
Human prostate cancer cells PC3 after 72 h of incubation in DMEM+	10–50 × 10^−6^M: 19 ± 11% (n.s.) proliferation	10–50 × 10^−6^M: 21 ± 9% (n.s.) proliferation	10–50 × 10^−6^M: 2 ± 2% (n.s.) proliferation
	10^−4^M = -58 ± 8% (*p* < 0.05) proliferation	10^−4^M = -38 ± 4% (*p* < 0.05) proliferation	10^−4^M = -7 ± 2% (n.s.) proliferation

Cell viability was evaluated by cellular dehydrogenase (DH) activity assay CCK8. Data are the mean ± SD, of nine replicates and expressed as a percentage (%) of DH, activity changes (Savino et al., 2018; Scala et al., 2019).

**FIGURE 4:**
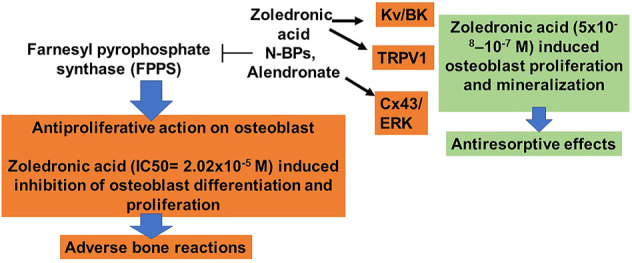
Dual actions of BPs on osteoblasts. Zoledronic acid-induced osteoblasts proliferation in the nanomolar concentration and activating ion channels and antiproliferative action in the micromolar concentrations. Also, nitrogen-containing BPs can inhibit FPP synthase, preventing cholesterol synthesis but at two hundred higher concentrations in respect to the osteoclast.

It should be of note that even if BPs can bind very strongly to bone mineral tissue, there is a continuous release of low levels of the drug. Consequently, there is a constant supply of BPs at very low concentrations, which may contribute to preserving osteoblast and osteocyte viability. Pro-survival effects are instead lost at high concentration, leading to a biphasic pattern of responsiveness of osteoblastic cells to the drugs (Figures 3, 4).

### The Action of Zoledronic Acid on TRPV1 and Kv/BK Channels

To evaluate the contribution of ion channels to the zoledronic acid-mediated proliferation and mineralization, the effects of the zoledronic acid used as a lead compound based on previous data (Savino et al., 2018) were tested in whole-cell patch-clamp experiments. Different cells types were tested such as preosteoclast-like cells RAW264.7, preosteoblast-like cells MC3T3-E1, and rat/mouse native bone marrow-derived mesenchymal stem cells (MSCs) (Scala et al., 2019). On RAW264.7, zoledronic acid (10 M–4 M) potentiated outward currents reduced by the Kv channel blocker tetraethylammonium hydrochloride (TEA) but not by the selective TRPV1-channel antagonist capsazepine. The involvement of the BK channel in the zoledronic acid-induced potentiation of the outward current cannot be excluded in these cells, since this channel plays a role in cell proliferation (Tricarico et al., 2002; Dinardo et al., 2012; Maqoud et al., 2018).

On pre-osteoblast MC3T3-E1 cells and bone marrow-derived MSCs, the observed zoledronic acid-evoked current (5 × 10^−8^ to 10^−4^ M) was instead reduced by capsazepine. On the other hand, the selective TRPV1-channel agonist capsaicin potentiated the control current supporting the functional role of this channel in this cell type. In the cell proliferation assays, 72 h incubation of RAW264.7 and MC3T3-E1 cells with zoledronic acid reduced proliferation, however with different potency. Indeed the IC_50_ values of 2.62 × 10^−7^ M and 2.02 × 10^−5^ M of this drug were respectively calculated for RAW264.7 and MC3T3-E1 cells (Table 1).

MC3T3-E1 cells and native murine bone marrow-derived osteoblasts undergo mineralization in the presence of capsaicin and sub-micromolar concentration of zoledronic acid (5 × 10^−8^–10^−7^ M). These effects were antagonized by capsazepine supporting the role of TRPV1 in this process. We concluded that the observed zoledronic acid-induced activation of the TRPV1 channel mediates the mineralization of osteoblasts and counterbalances the anti-proliferative effects linked with hFPPS inhibition at higher concentrations. While, osteoclasts lack the zoledronic-induced activation of TRPV1 channel with cell death (Scala et al., 2019) (Figure 3).

The interaction of zoledronic acid with TRPV1 ion channels was evaluated by testing its effects in patch-clamp experiments on TRPV1-transfected *Xenopus laevis* oocytes (inside-out configuration) and HEK293 cells (whole-cell configuration). Also in oocytes this drug was found to act as an agonist of TRPV1 ion channel (Scala et al., 2019).

**FIGURE 5:**
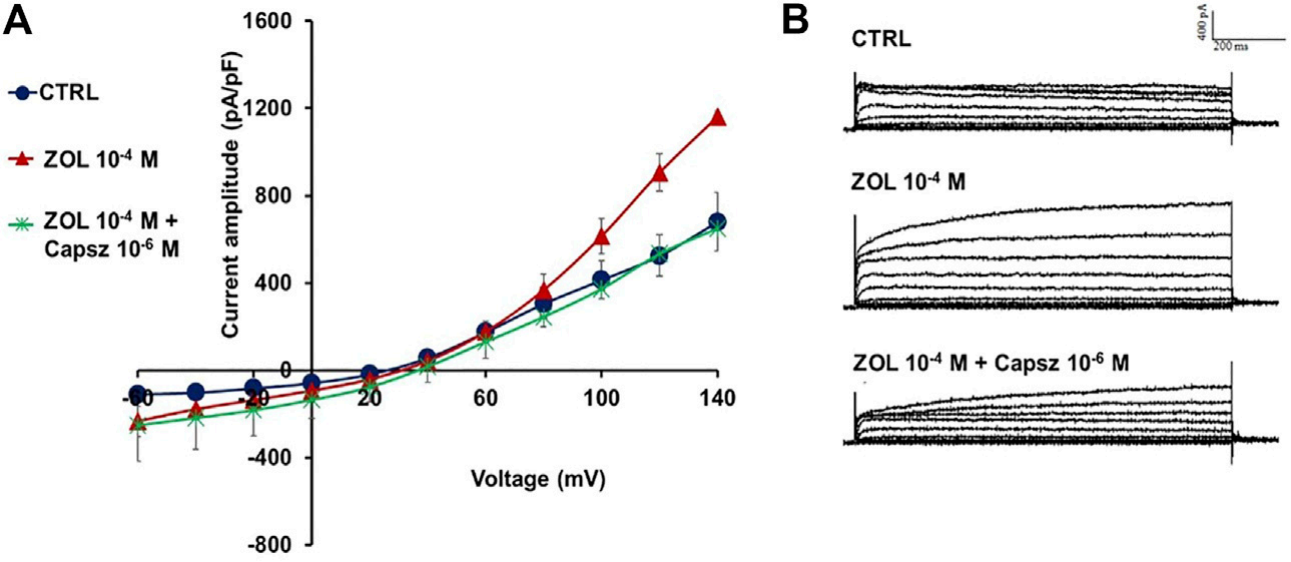
Zoledronic acid (ZOL) effects on TRPV1 channel on neuronal SH-SY5Y cells. **(A)** Macroscopic currents induced by ZOL (10^−4^ M) using a whole-cell configuration on SH-SY5Y neuronal cells. Capsazepine (Capsz) completely closed ZOL-evoked currents. Current was obtained in response to voltage pulses from - 60 mV to +140 mV in 20 mV steps, starting from HP = -60 mV (Vm). Cells characterized by the same size were selected for patch-clamp experiments. Each point represented the mean ± SEM (N patches = 5–7). Temperature: 21 ± 1 °C. **(B)** Sample traces of control (CTRL) current, ZOL-evoked and ZOL + Capsz-evoked instantaneous current.

We recently showed that zoledronic acid (ZOL) (10^−4^ M) may potentiate TRPV1 channel on neuronal SH-SY5Y cells, both at negative and positive voltages; at positive membrane voltages, ZOL-evoked currents were almost completely eliminated after the application of capsazepine, supporting the idea that ZOL can activate TRPV1 ion channels in neuronal cells (Figure 5) (Scala et al., 2020a).

### The Action of Zoledronic Acid on KATP Channels

**FIGURE 6:**
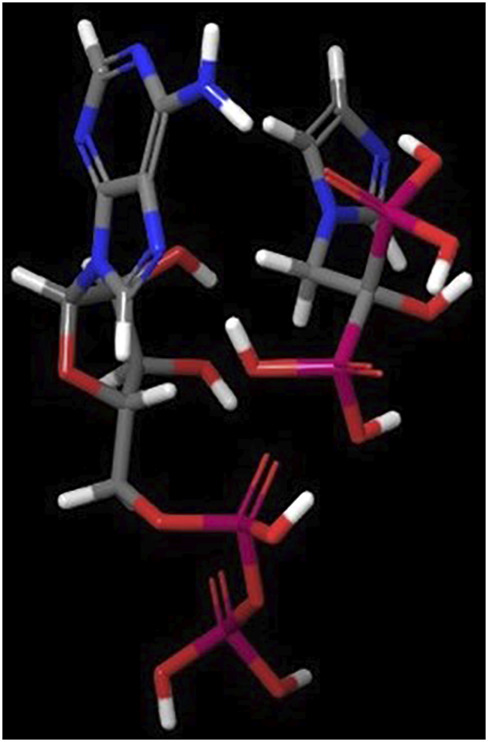
Zoledronic acid is a nitrogen-containing molecule whose structure shows similarities also with nucleotides. Blue colour = Nitrogen atom, Grey = Carbon atom, White = Hydrogen atom, Red = Oxygen, Purple = Phosphate groups. Left-hand ADP and right-hand zoledronic acid molecules, respectively.

The zoledronic acid is a nitrogen-containing molecule whose structure shows similarities also with nucleotides, endogenous ligands of ATP-sensitive K^+^(KATP) channels (Figure 6). We recently discovered the action of this drug on different subtypes of KATP channels (Maqoud et al., 2021). Zoledronic acid was a potent blocker of the Kir6.1-SUR2B and Kir6.2-SUR2A channels inhibiting the whole-cell KATP channel current of recombinant Kir6.1-SUR2B and Kir6.2-SUR2A subunits expressed in HEK293 cells with an IC_50_ of 3.9 ± 2.7×10^–10^ M and 7.1 ± 3.1×10^–6^ M, respectively, and the native channels currents recorded in osteoblasts, slow-twitch (Soleus) and fast-twitch (Extensor digitorum longus) skeletal muscle fibers. The rank order of potency that we observed based in our experiments performed on recombinant and native channels in inhibiting the KATP currents was: Kir6.1-SUR2B/Soleus-KATP/osteoblast-KATP > Kir6.2-SUR2A/Extensor digitorum longus-KATP >>> Kir6.2-SUR1 and Kir6.1-SUR1 (Scala et al., 2020b; Maqoud et al., 2021).

“In silico” studies revealed that the drug binds to the ADP/ATP sites on Kir6.1-SUR2B and Kir6.2-SUR2A and on the sulfonylurea pocket on SURs (Maqoud et al., 2021). Therefore, we concluded that zoledronic acid interacts with the channels composed of Kir6.1/6.2 and the SUR2A/B subunits with high affinity compared to the channels formed of Kir6.2 and SUR1 mimicking the pancreatic beta cells channel.

Additionally, zoledronic acid’s inhibitory action can be indirectly mediated by isopentenyl diphosphate that accumulates in the cells following the zoledronic acid-dependent inhibition of the hFPPS that it is converted to ATP isopentenyl ester. These toxic molecules inhibit Kir6.1/2-SUR2 protein complexes with high specificity at the corresponding ATP binding regions, with ATP isopentenyl ester being found also to inhibit the ADP/ATP carrier. This has important implications for mitochondrial function and ADP/ATP carrier-mediated mitochondrial apoptosis triggering and could represent an additional zoledronic acid-mediated indirect mechanism of blocking KATP channels (Maqoud et al., 2021).

The interaction of zoledronic acid with multiple binding sites on the Kir6.1/2 and SUR2B/A and the interaction of other ligands released into the cytosol help to explain the potency of this drug in soleus fibers and osteoblast cells.

Cantu’ syndrome (CS) is a rare autosomal dominant inheritance, multi-organ condition characterized by cardiomegaly, vascular dilation, and low blood pressure (Nichols et al., 2013), hypertrichosis, neuromuscular symptoms, and skeletal malformations. The molecular basis of CS is based on the GOF mutations in the *ABCC9* and *KCNJ8* genes encoding for the SUR2 and Kir6.1 subunits, respectively, (Harakalova et al., 2012), of KATP channels. To date, >70 individuals with CS, associated with >30 missense *ABCC9* or *KCNJ8* mutations have been reported. The human patients affected by CS with a gain of function mutations (GOF) mutations of *ABCC9* and *KCNJ8* genes show musculoskeletal abnormalities including fatigue and abnormal bone morphology with facial dysmorphism. The bone marrow cells from Kir6.1 (V65M) and the SUR2 (A478V) CS mice following differentiation failed to mineralize in our hands suggesting of abnormal mineralization process driven by the KATP channels. Histopathological analysis of femora bone sections from these mice, however, did not reveal severe abnormalities like those observed for instance in the skeletal muscles (Scala et al., 2021; Scala et al., 2020a) of the same animals, unless some osteoblasts aggregate were observed in some sections. We have demonstrated that CS mutation in the *KCNJ8* genes encoding for the Kir6.1WT/VM is responsible for severe injuries in the skeletal muscle of a murine model of CS, reporting how GOF mutation caused atrophy, necrosis, and loss of muscle with fibrotic replacement, inflammatory cell infiltration, up-regulation of autophagy genes, and reduced muscle strength in the slow twitching muscle and impairment of glibenclamide response (Scala et al., 2020b). We also reported that in SUR2WT/AV and SUR2AV/AV mice, forelimb strength was reduced associated with loss of modulation of KATP channels by MgATP with metabolic decoupling and atrophy in different muscles (Scala et al., 2021). A slight rightward shift of sensitivity to inhibition by glibenclamide was detected in SUR2AV/AV mice indicative however of a conserved response to this drug.

Glibenclamide is proposed in these CS patients. This drug concentration-dependently reduces the KATP channel current of mutants hetero-homozygous SUR2 expressed in a heterologous expression system, and in cardiac, smooth, and skeletal muscles cells but is much less effective against hetero-homozygous Kir6.1 (V65M) mutation (Huang et al., 2018; Scala et al., 2020b). The *ABCC9*-transgenic mice are in use for testing the effects of the chronic administration of glibenclamide that is capable to reverse the vascular and cardiac phenotypes (Ma et al., 2019; McClenaghan et al., 2020). These data suggest that a high-dosing protocol of glibenclamide under glycemia monitoring can be effective in patients carrying the *ABCC9* mutations, but *KCNJ8* CS patients may not benefit from this treatment option.

CS patients report also a large variety of bone malformations, some CS patients may suffer from bone frailty. For this purpose, we are investigating the action of zoledronic acid on KATP channel currents of skeletal muscle fibers and bone cells from CS mice to evaluate the potential use of this drug in CS.

## Discussion

Bisphosphonates modulate ion channels through the intracellular accumulation of DMAPP, IPP, FPP, and ATP derivatives like the isopentenyl diphosphate that accumulate in the cells following the zoledronic acid-dependent inhibition of the FPPS as we also observed (Maqoud et al., 2021). An emerging mechanism of action of this drug is the direct interaction of zoledronic acid with channel subunits like KATP (Kir6.1, Kir6.2, SUR2, SUR1) and TRPV1 channels. Zoledronic acid behaves as an agonist of the TRPV1 channel. However its mechanism of action needs to be investigated at single-channel level. Indeed, the action of zoledronic acid can be limited by factors including its poor permeability through the membrane, the release of the intermediate molecules, and the existence of conformers. The zoledronic acid-induced activation of the TRPV1 explains the zoledronic-induced mineralization of osteoblasts and its pain-relieving effects. Zoledronic acid is commonly used to treat several bone diseases, like osteoporosis and Paget’s disease. Despite of belonging to WHO’s List of Essential Medicines, many aspects linked to the pharmacological effects of zoledronic acid are still unclear. For example, empirical evidence has shown that zoledronic acid may exert pain-relieving effects in the presence of different noxious stimuli. At the same time, a recent FDA alert has reported an increased risk of bone pain and muscle aches in patients taking BPs, showing disabling pain conditions. The molecular pathways involved in zoledronic acid-mediated pain are unknown in both cases. Among the proteins involved in pain sensation, the TRPV1 ion channel is universally recognized to play a key role. The selective channel agonist capsaicin is commonly used in fibromyalgia and joint conditions, such as osteoarthritis. We have recently demonstrated that zoledronic acid is an agonist of TRPV1 ion channel in bone cells, being able to activate strong outward capsazepine-sensitive currents on pre-osteoblast like cells MC3T3-E1 and native murine/rat mesenchymal stem cells. Zoledronic acid also increases TRPV1 currents in neuronal SH-SY5Y cells as well as the TRPV1 current carried by the recombinant TRPV1 subunit expressed in a cell line (Scala et al., 2019).

Nitrogen-containing bisphosphonates like zoledronic acid showed reduced analgesic response vs. the non-nitrogen bisphosphonates such as etidronate and clodronate in animal models of hyperalgesia. Etidronate and clodronate displayed their analgesic effects at doses lower than those leading to the anti-bone-resorptive effects. Analgesic effects of these drugs evaluated in the abnormal writhing responses in mice associated with the acetic acid-induced neuronal expression of c-Fos and the elevation of blood corticosterone, and the hind-paw licking/biting response induced by intraplantar injection of capsaicin, but not by the intraperitoneal injection of 1% acetic acid (Kim et al., 2013). The reduced efficacy of the nitrogen-containing bisphosphonates in these animal models of pain can be explained by the activation of the TRPV1 pathway.

**FIGURE 7:**
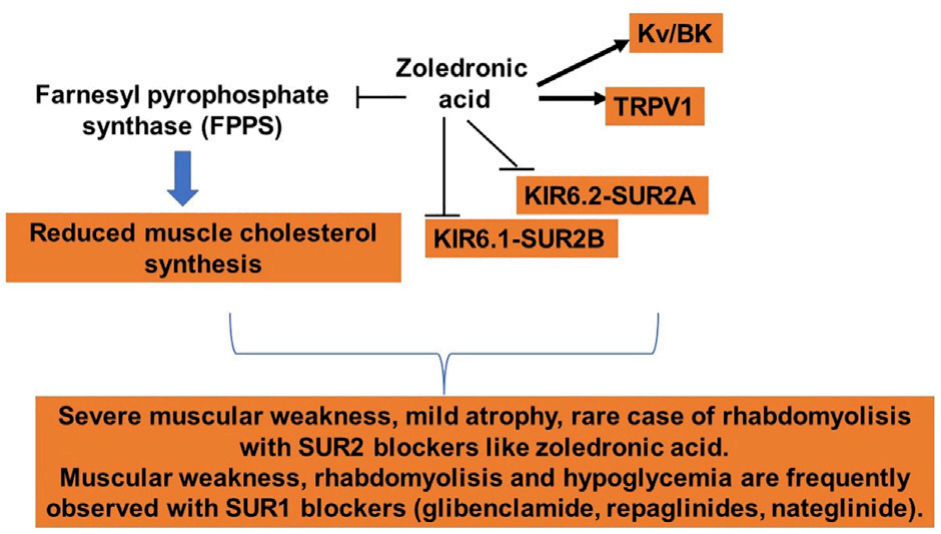
Zoledronic acid actions in skeletal muscle. The drug inhibits the farnesyl pyrophosphate synthase leading to reduced cholesterol production, potentiates the TRPV1 and BK channels, and inhibits the KATP channels more specifically the channel composed by the Kir6.2-SUR2A, but also inhibit the Kir6.1-SUR2B channel located in the soleus muscle and the microvasculature.

The interaction of zoledronic acid with KATP channels subunits that we observed “*in vitro*” and “in silico” is correlated with the skeletal muscle adverse drug reactions that together with the bone reactions were the most frequently reported A.D.R. for this drug in the EudraVigilance database (Maqoud et al., 2021) (Figure 7). Multiple correlation analyses showed that muscular weakness and rhabdomyolysis Proportional Reporting ratio (P.R.R.) were correlated with the IC50 of the sulfonylureas, glinides, and zoledronic acid to block the recombinant KIR6.2-SUR2A channel currents expressed in cell lines mimicking the main KATP channel subtype in skeletal muscle. It is of note that rare cases of rhabdomyolysis were reported for zoledronic acid with P.R.R. <1 vs. the higher prevalence of these reactions for the glibenclamide, repaglinide, and nateglinide with P.R.R. > 1 (Figure 7) (Maqoud et al., 2021).

We also found a significant correlation between the combined atrial fibrillation and arrhythmias reactions and the IC_50_ to block either the Kir6.2-SUR2A and Kir6.1-SUR2B channels mimicking the cardiac and vascular KATP channels, respectively. The atrial fibrillation and hypertension reactions were also associated with the Kir6.1-SUR2B blocking actions (Maqoud et al., 2021) and are a common and limiting reaction with long-term therapy with zoledronic acid. Unfortunately, we currently failed to find any statistical correlation between the P.R.R. of the osteonecrosis of the jaw, the most frequent bone reaction of bisphosphonate and myalgia, and the ability of zoledronic acid to block KATP channels. Despite this unexpected result, it may be possible that an indirect mechanism of interaction mediated by ATP nucleotide analogs plays a role in inhibiting the Kir6.1-SUR2 channel complex exerting vasoconstriction of the micro-vessels and osteoblast cell disruption.

The action of zoledronic acid and the KATP channel blockers in skeletal muscle are some-how anticipated by the findings that the *in vivo* down-regulation of KATP channel subunits in an animal model of atrophy. More specifically, the SUR1 subunit and the *in vitro* pharmacological blockade by the glibenclamide and repaglinide or by the tyrosine kinase inhibitor staurosporine are coupled to atrophic signaling in skeletal muscle, and these cytotoxic actions are prevented by the KATP channel opener diazoxide (Tricarico et al., 1997, 1998, 2010; Mele et al., 2012, 2014a, 2014b; Cetrone et al., 2014) (Figure 7).

TRPV1 can be a target of zoledronic acid in skeletal muscle although its function is not known in this tissue. We recently found that the TRPV1 gene is upregulated in the skeletal muscle of an animal model of metabolic stress parallel to the oxytocin receptor gene preserving animal health, suggesting cytoprotective action of this gene (Conte et al., 2021).

It should be noted that the administration of 4 mg of zoledronic acid per e. v., reached a peak within 15 min from the infusion and declined rapidly to a concentration <10% of the Cmax after 4h <1% of the Cmax after 24 h, followed by a prolonged period of very low concentration of about 0.1% of the Cmax until the second infusion generally at 28 days from the first infusion. After the first 24 h, 39 ± 16% of the administered dose is present in the urine, while the remaining part is mainly linked to bone tissue (ZOMETA^®^). It has been shown that following a single e. v. dose administration of 4 mg to female patients for osteoporosis treatment, the free plasmatic concentrations of zoledronic acid were 1.5 mol/L × 10-6 mol/L after 1 h, 3.6 mol/L × 10-7 mol/L, and 2.46 mol/L × 10-8 mol/L respectively after 4 and 24 h from the first infusion (Veldboer et al., 2011). These findings suggest that zoledronic acid may exert acute cytotoxic action within 4 h from the first infusion unless renal insufficiency enhances the zoledronic acid levels while exerting anabolic action interacting with ion channels on osteoblasts in the long term. The cumulative effects are expected since the drug is released from the bone tissue very slowly into the systemic circulation and then eliminated by the kidney with a body clearance of 5.04 ± 2.5 L/h, regardless of dose and not affected by sex, age, race, and body weight (ZOMETA^®^).

## Conclusion

Ion channels are emerging targets and anti-target for bisphosphonates. Zoledronic acid can be the first selective musculoskeletal and vascular KATP channel blocker available, targeting the Kir6.1-SUR2B and the Kir6.2-SUR2A subunits with high affinity. The action of this drug against the CS mutants may improve the appropriate prescription in those CS patients affected by musculoskeletal disorders such as bone fracture associated with bone frailty. We do not know if the CS patients may benefit from the zoledronic acid since the limited drug distribution in the skeletal muscle apparatus may limit its efficacy in resolving the cardiac abnormalities observed in CS.
